# Au nanostructure fabrication by pulsed laser deposition in open air: Influence of the deposition geometry

**DOI:** 10.3762/bjnano.8.242

**Published:** 2017-11-17

**Authors:** Rumen G Nikov, Anna Og Dikovska, Nikolay N Nedyalkov, Georgi V Avdeev, Petar A Atanasov

**Affiliations:** 1 Institute of Electronics, Bulgarian Academy of Sciences, 72 Tsarigradsko Chaussee, Sofia 1784, Bulgaria,; 2 Rostislaw Kaischew Institute of Physical Chemistry, Bulgarian Academy of Sciences, Acad. G. Bonchev Str., Bl.11, 1113 Sofia, Bulgaria

**Keywords:** Au nanostructures, deposition geometry, nanocolumns, open-air PLD, physical properties

## Abstract

We present a fast and flexible method for the fabrication of Au nanocolumns. Au nanostructures were produced by pulsed laser deposition in air at atmospheric pressure. No impurities or Au compounds were detected in the resulting samples. The nanoparticles and nanoaggregates produced in the ablated plasma at atmospheric pressure led to the formation of chain-like nanostructures on the substrate. The dependence of the surface morphology of the samples on the deposition geometry used in the experimental set up was studied. Nanocolumns of different size and density were produced by varying the angle between the plasma plume and the substrate. The electrical, optical, and hydrophobic properties of the samples were studied and discussed in relation to their morphology. All of the nanostructures were conductive, with conductivity increasing with the accumulation of ablated material on the substrate. The modification of the electrical properties of the nanostructures was demonstrated by irradiation by infrared light. The Au nanostructures fabricated by the proposed technology are difficult to prepare by other methods, which makes the simple implementation and realization in ambient conditions presented in this work more ideal for industrial applications.

## Introduction

The growing interest in nanomaterials is related to their unique and fascinating properties [1–2], which are not observed in their bulk counterparts. Moreover, the strong dependence of the physical and chemical properties of these structures on their surface morphology makes them very attractive for a wide range of applications [3–9]. Initially, the nanofabrication process has involved the use of expensive and time-consuming technologies. Over the years, the different requirements for the characterization of the properties of the nanostructures and the methods for their fabrication have established different subfields in nanotechnology. One subfield is devoted to the development of inexpensive, easy-to-use, flexible and time-saving nanofabrication techniques. The efforts of the other scientific groups have been directed to producing nanostructures for applications in areas such as nanomedicine that impose specific requirements regarding the technology behind their fabrication. Such applications require contamination-free nanostructures, suggesting that the development and use of physical nanofabrication methods is further warranted.

One of the physical vapor deposition techniques widely applied in bottom-up nanotechnology is pulsed laser deposition (PLD). Initially, PLD technology was used mainly for deposition of thin films with a high crystallinity, accurate stoichiometry, and thickness control on the order of an atomic monolayer [10]. In recent years, this method has been increasingly applied to growing nanostructures with properties that depend on the deposition conditions [11–13]. The possibility to control the morphological and structural characteristics of the ultimate structure by precisely manipulating the experimental parameters makes PLD one of the most promising techniques for formation of complex oxide heterostructures and nanostructures [14–16]. Over the years, the realization of the PLD set up in oblique angle deposition (OAD) geometry has also been developed [16]. It has been demonstrated that a variety of structures arranged as nanocolumns with different sizes and densities could be produced by application of this specific geometry [17–18].

Despite the attractive properties and practical advantages of PLD, there still exist some drawbacks and limitations in using this method. The PLD process is typically performed in a vacuum chamber at ultrahigh vacuum or in the presence of a background gas, such as oxygen, nitrogen or argon. Therefore, the need for a specific environment limits the flexibility of the method, which hampers its efficient application in the industry. Recently, several research groups have succeeded in overcoming this limitation through the implementation of laser deposition in air at atmospheric pressure (in open air) [19–22]. Boutinguiza et al. have shown that it is possible to produce Ag nanoparticles by this approach [20]. A practical application of the method has already been demonstrated, namely, the formation of hard coatings [22]. PLD in open air could also lead to the formation of highly porous structures on different substrates [19,21]. Our previous results showed that the morphology of gold structures produced by PLD in open air depends on the target–substrate distance, laser fluence, number of laser pulses applied, and the laser wavelength used [23–24]. Furthermore, a decrease in the target–substrate distance below 3 mm resulted in the formation of denser, larger structures compared to deposition at larger distances [23]. In the latter case, micrometer-sized droplets and fine nanowires around and over them were obtained [21,23]. Despite the recent significant progress in the field, the influence of the deposition geometry of PLD in open air on the surface morphology of the structures produced has yet to be thoroughly studied. The nanostructures produced by laser deposition in air are still being investigated to establish their physical properties and possibilities for application.

In the present paper, we report the results on the fabrication of Au nanostructures by PLD in open air. The influence of the PLD deposition geometry on the surface morphology and physical properties of the samples was studied. The structures formed are columns comprised of an ensemble of nanoparticles, structures that have not been previously fabricated. The optical, electrical and hydrophobic properties of the samples produced were studied and discussed in relation to their morphology. The modulation of the electrical properties of the Au nanostructures by irradiation by infrared light was demonstrated.

## Experimental

The fabrication of the Au nanostructures was implemented in a one-step deposition of the ablated material on a substrate. The Au target (purity of 99.99 %) was mounted on a rotating holder in order to prevent deep drilling during laser irradiation. The ablated material was deposited on quartz substrates. The laser ablation was performed using an Nd:YAG laser system (Lotis LS-2147) operating at a wavelength of 355 nm with a pulse duration of 15 ns and a repetition rate of 10 Hz. The PLD experiments were carried out in air at atmospheric pressure. The angle between the incident radiation and the target surface was about 30°. This resulted in an elliptical spot on the target surface. The configuration of the substrate and the target was changed in order to study the influence of the laser spot shape on the morphology and physical properties of the structures produced. Four geometries were considered in the experiments, as presented in Table 1. In geometry 1, 2, and 3, the angle between the plasma plume and the substrate was fixed at 5°. In geometry 1, the substrate was placed parallel to the long axis of the laser spot. In geometry 2, the substrate was fixed at an angle of 45° with respect to the spot’s long axis. In geometry 3, the substrate was perpendicular to the spot’s long axis. In geometry 4, the substrate and the target were parallel (standard on-axis configuration). The target–substrate distance was fixed at 2 mm. The laser fluence used was 18 J/cm^2^ and the number of laser pulses was 1800 for all samples. The morphology of the structures produced was observed by scanning electron microscopy (SEM) (LYRA Tescan). Au samples were also deposited in open air on transmission electron microscopy (TEM) grids using a shorter deposition time (600 pulses) in order to investigate the structure of the material ablated. The TEM and selected area electron diffraction (SAED) images were taken on a HR scanning transmission electron microscope (STEM) (JEOL JEM 2100) to reveal the morphology and crystallinity of the as-deposited samples. For STEM measurements, a drop of distilled water was placed on the sample and material from within the drop was removed by scratching. The drop with the removed material was collected by a pipette and transferred onto a TEM grid. The crystalline structure of the samples was analyzed by X-ray diffraction (XRD). Goniometric scans for phase identification were recorded in the 2θ interval of 20–80° using a Philips PW 1050 diffractometer equipped with a Cu Kα tube and a scintillation detector. The composition of the material deposited on the substrate was determined by X-ray photoelectron spectroscopy (XPS) by means of an AXIS Supra electron spectrometer (Kratos Analytical Ltd.). The thickness of the samples was measured using an optical profilometer (Zeta Instruments). The optical properties of the structures produced were estimated based on the transmission spectra taken using an optical spectrometer (Ocean Optics, HR 4000). The resistivity of the samples was measured using the four-point probe method. The electrical response of the samples was studied during irradiation by a 30 mW CW laser operating at 785 nm. Also, the hydrophobic properties of the samples were tested by measuring the contact angle of a water drop (*V*_d_ = 2 μL) with respect to the underlying substrate surface.

**Table 1:** Deposition geometries used in the experiments.

	Geometry 1	Geometry 2	Geometry 3	Geometry 4

Side view	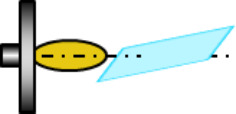	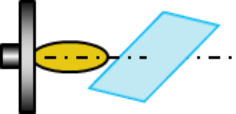	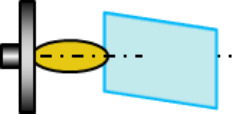	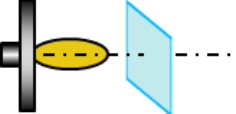
Front view	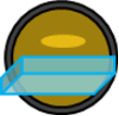	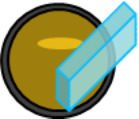	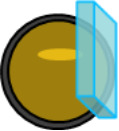	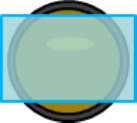

## Results and Discussion

### Nanostructure formation

We present the general aspects of the nanostructure fabrication process using geometry 4 as an example, where the samples were deposited using a standard on-axis configuration. Typically, the deposition process of Au in open air led to the formation of a black spot on the transparent substrate. The diameter of the darkest area was 3–4 mm, with concentric color fading visible outside the central spot. A larger fabrication area could be achieved by scanning the target surface with the laser beam or by the relative movement of the substrate with respect to the target.

**Figure 1:**
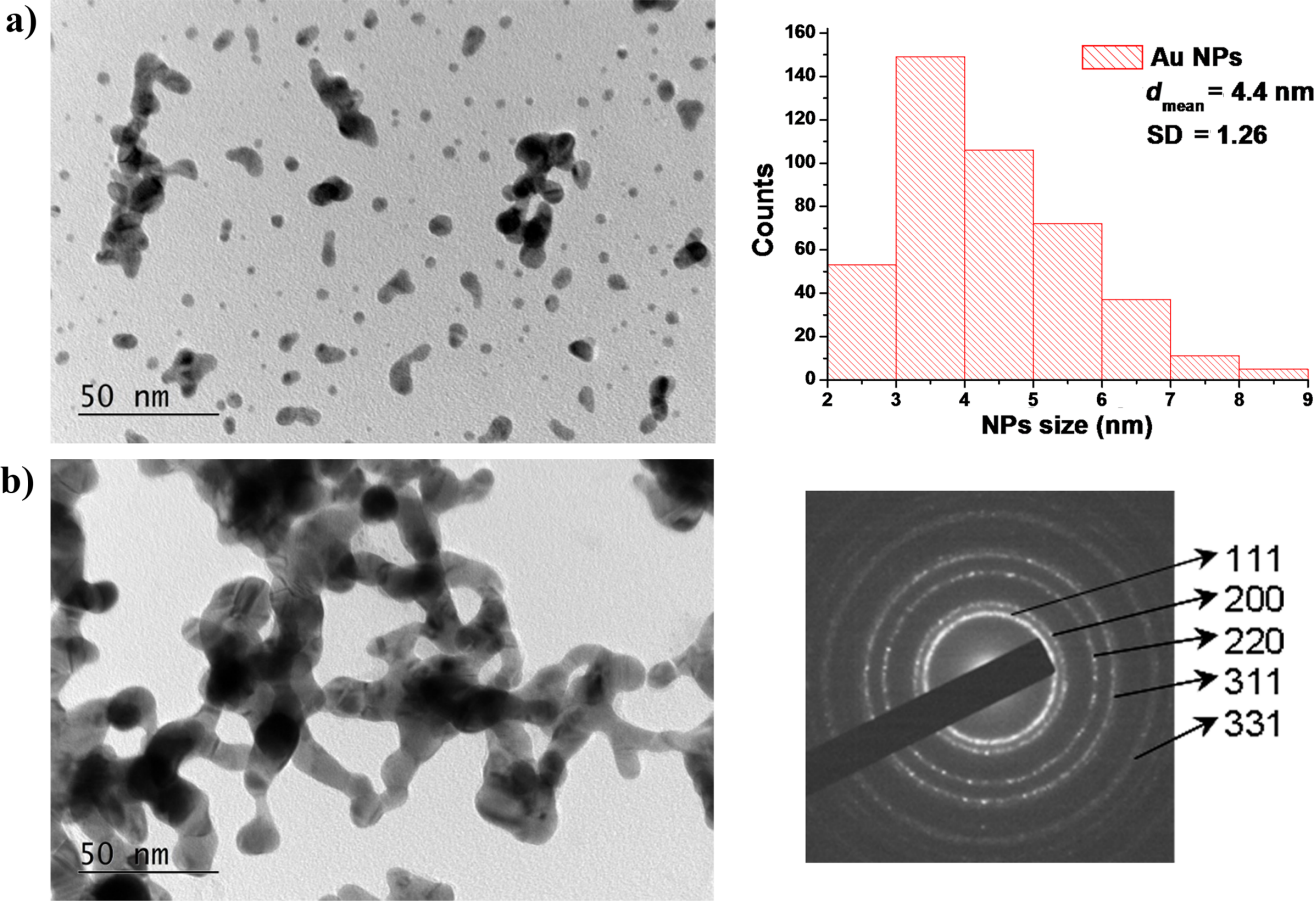
(a) TEM image of the material ablated in open air and the size distribution of the nanoparticles produced using 600 laser pulses. (b) TEM and SAED images of the Au sample deposited using 1800 laser pulses on a quartz substrate and transferred to a TEM grid. The samples were deposited in a standard on-axis configuration (geometry 4).

To clarify the mechanism of formation of the nanostructures obtained under the conditions presented above, deposition at shorter times was performed. A TEM image of the material ablated in open air is presented in Figure 1a. As can be seen, the ablated material consists of individual nanometer-scale particles and aggregates of different size and shape. The density of the ablated material on the TEM grid is low and the overlapping of the nanoparticles is negligible due to the short deposition time. An experiment using the same processing conditions but performed in vacuum led to the deposition of a flat film. Furthermore, the room substrate temperature does not support an efficient atom migration. All these factors suggest that the nanoparticles, as well as the nanoparticle aggregates, are predominantly formed in the ablation plume due to intensive collisions between the particles, which confirms the previous results [21]. The nanoparticle size distribution corresponding to the TEM image is also shown in Figure 1a. The diameter of the nanoparticles ranges from 2 to 9 nm and the mean diameter is around 4 nm. All the particles and agglomerates are crystalline, which is confirmed by the SAED pattern (not shown). A TEM image of the material deposited using 1800 laser pulses on the substrate is presented in Figure 1b. The image is of the material transferred from the substrate to the TEM grid according to the procedure described in the Experimental section. The nanoparticles and aggregates produced in the ablated plasma led to the formation of a chain-like nanostructure on the substrate. SAED electron diffraction patterns of the nanostructure are also shown in Figure 1b. The interplanar distance from the main reflections could be assigned to a family of planes of metallic Au (Au cubic, S.G. Fm3m, *a* = 4.0786 Å, PDF 04-0784).

**Figure 2:**
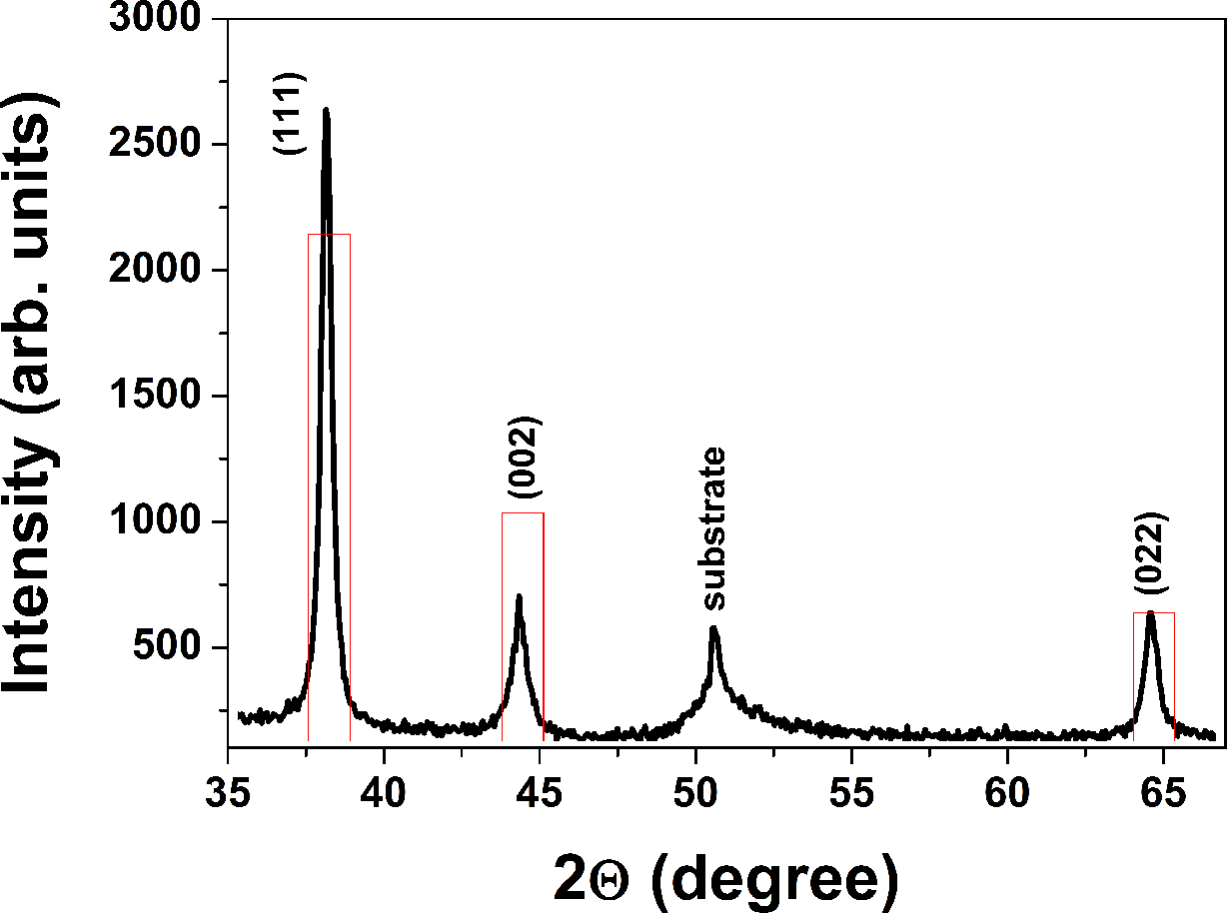
XRD pattern of a Au nanostructure produced by PLD in open air under standard on-axis configuration (geometry 4) compared with theoretically calculated XRD patterns of Au (red columns).

An XRD pattern of the Au sample is shown in Figure 2. The XRD spectrum reveals diffraction peaks corresponding to particular planes of cubic Au, such as (111), (002), and (022) (ICDD: 98-061-1625). For face-centered cubic metals, like Au, the most common crystallographic texture appears in the [111] direction. We observed a weak texture in the same direction, which is presented in Figure 2, where the experimental diffraction pattern is compared with a calculated randomly oriented powder pattern with intensity normalized by the (022) peak. On the other hand, as was previously reported, the same structures were obtained independently on crystalline or amorphous substrates [21]. This gives us a reason to suggest that the crystalline nanoparticles and aggregates formed in the ablation plume are arranged along the (111) reflection plane of Au, which possesses the lowest surface-free energy. This crystallite alignment leads to the chain-like nanostructure formation on the substrate, as shown in Figure 1b.

**Figure 3:**
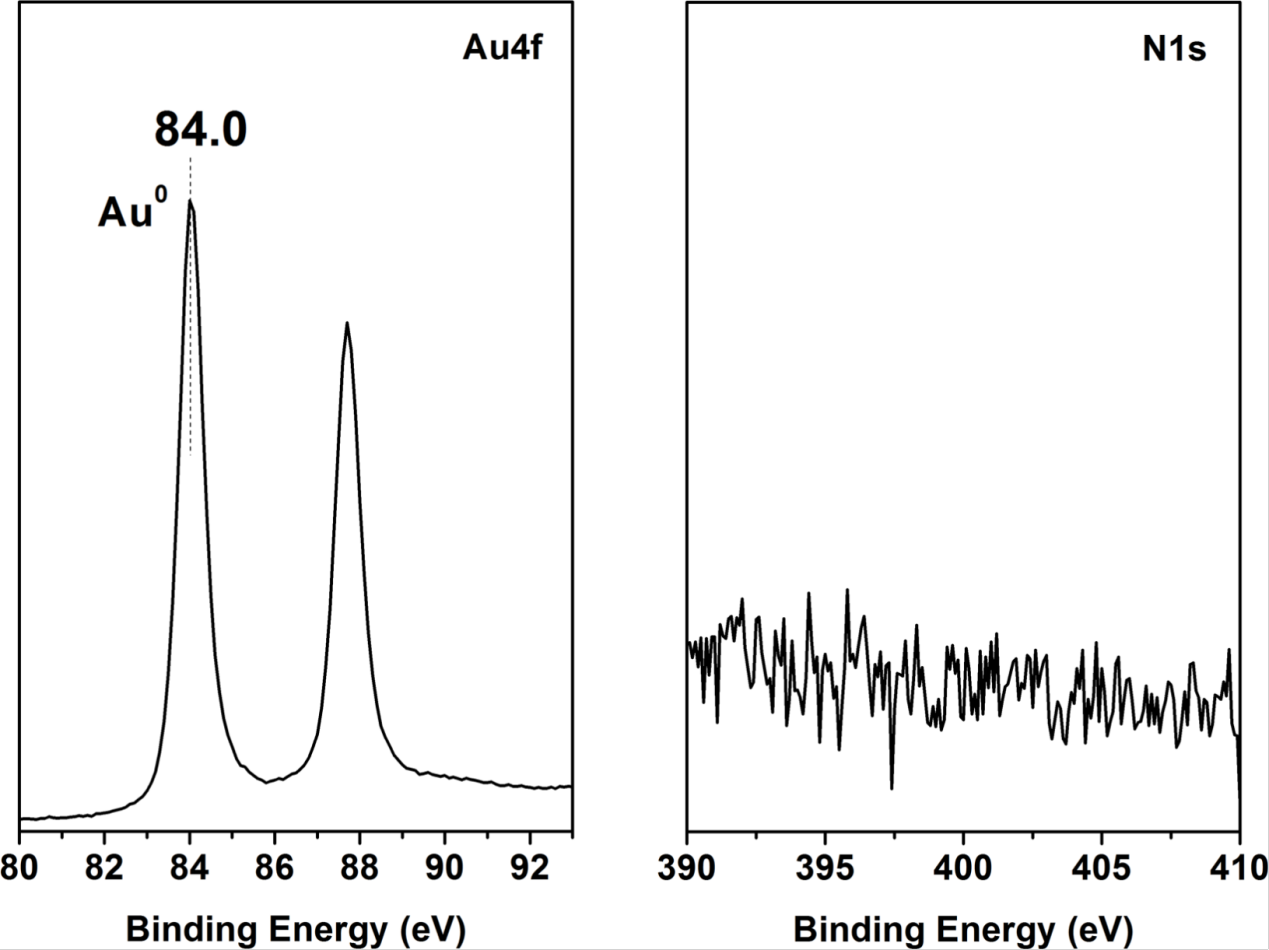
XPS spectra of Au nanostructures produced by PLD in open air in a standard on-axis configuration (geometry 4).

The XPS analysis of the as-deposited Au nanostructure is presented in Figure 3. The presence of Au was confirmed by recording the characteristic Au 4f peak at a binding energy of 84.0 eV corresponding to bulk metallic gold. No nitrogen or oxygen inclusions were detected. This result gives us reason to conclude that the PLD in open air is able to produce pure Au samples without any impurities or Au compounds. Furthermore, the structures produced are complex 3D ensembles of nanoparticles.

### Influence of the deposition geometry

**Figure 4:**
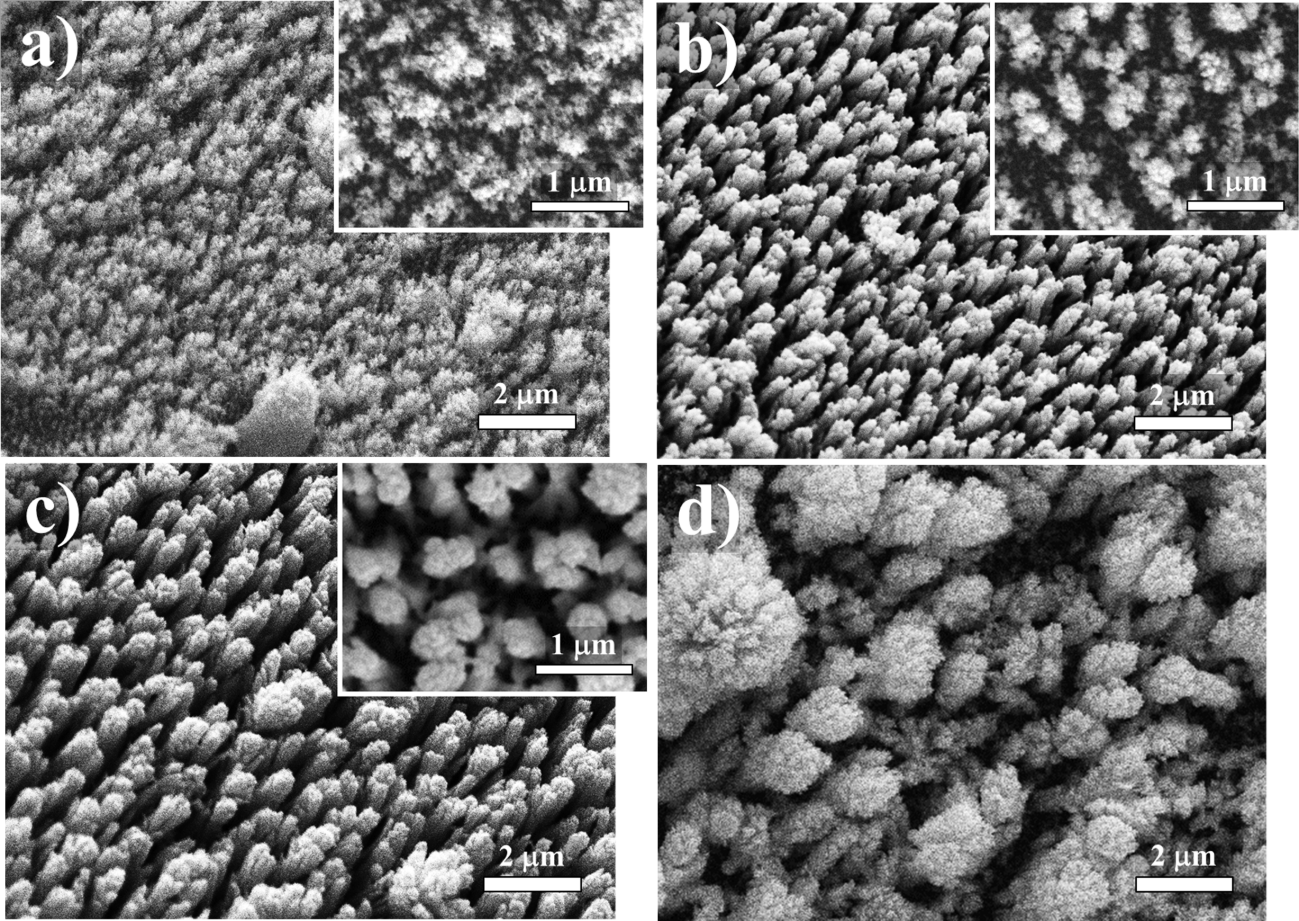
SEM images of the Au nanostructures deposited using (a) geometry 1, (b) geometry 2, (c) geometry 3, and (d) geometry 4. The insets show a top view of the structures.

The change of the substrate orientation with respect to the plasma plume allows different deposition geometries to be implemented. Figure 4 shows the SEM images of the surface morphology of the samples deposited at different geometries. As can be seen, the deposition process resulted in discrete structures, rather than of a flat film. The structure of the samples produced using geometry 1, 2 and 3 consisted of nanometer-sized columns. The nanocolumn production is due to the effect of geometric shadowing – when the deposition takes place at small angles, the incoming material flux only lands on the taller parts of the growing structure [25–26]. It is well known that the size and density of the nanocolumns are a function of the angle between the material and the substrate [25,27]. Since in our experiments the laser radiation was incident to the target at a small angle, the resulting spot had an elongated shape, which led to the formation of a plume with a highly asymmetric cross-section [28]. Consequently, the efficiency of deposition and the material shadowing effect depended on the position of the substrate with respect to the plasma plume. The nanocolumns with the highest density were obtained using deposition geometry 1 (Figure 4a). The dense covering of the substrate (estimated at 85%) here was also accompanied by the formation of a finer structure (≈100–250 nm column diameter) in contrast to those obtained at the other geometries, which can be seen in the top view of the samples (insets in Figure 4). The deposition using geometry 2 produced nanocolumns with diameters ranging from 100 nm up to ≈400 nm (Figure 4b). The structures with a larger nanocolumn diameter and a lower density (56% coverage) observed here are related to the more efficient material shadowing when using this set up compared to the deposition under geometry 1. The use of geometry 3 resulted in an even more pronounced shadowing effect, resulting in nanocolumns with diameters in the range ≈200–600 nm (66% coverage), as shown in Figure 4c. For on-axis PLD configuration (geometry 4), the deposition process led to the formation of distinct features with a characteristic size in the range from 700 nm to 3 µm (79% structure coverage) comprised of an ensemble of smaller particles (Figure 4d). In this case, the deposition of the nanoparticles, aggregates and droplets, and their growth by accumulation of material, were the main mechanism driving the nanostructuring process. It could be concluded that the morphology of the structures obtained by PLD in open air depends strongly on the orientation of the substrate with respect to the target.

It should be mentioned that fabrication of Au nanostructures by physical methods has rarely been presented in the literature. The formation of Au nanocolumns with controllable characteristics is difficult to achieve due to the high mobility of gold [29].

### Physical properties of the samples

**Figure 5:**
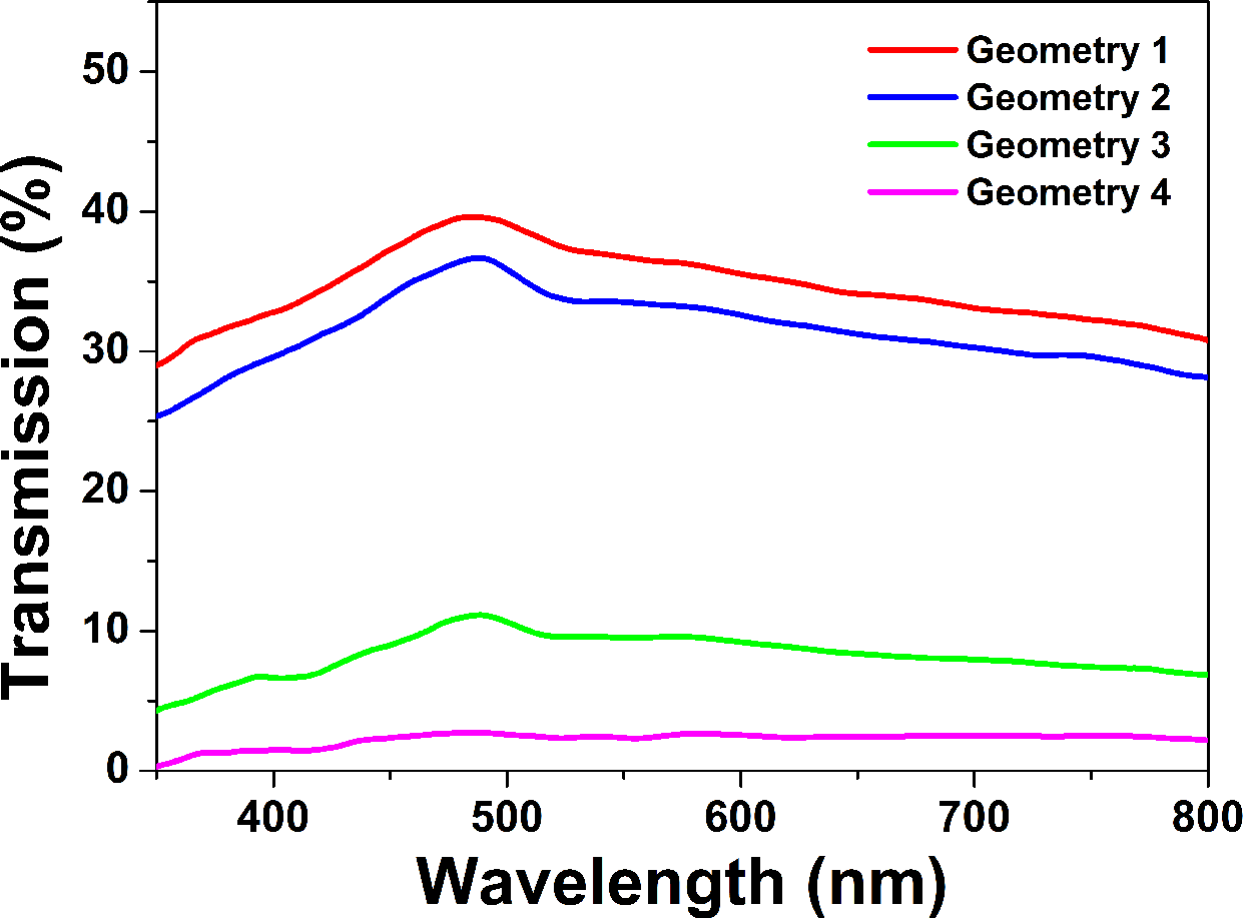
Optical transmission spectra of the Au nanostructures deposited at different geometries.

Figure 5 shows the optical transmission spectra of the samples produced at different PLD geometries. As can be seen, the transmission is characterized by a difference in intensity in the spectral range presented. The difference observed is related to the different morphology (dense covering of the substrate) and thickness of the structures. In principle, one of the main drawbacks of the PLD method is a nonuniform thickness distribution of the ablated material deposited on the substrate. The difference in the film thickness (central part and periphery of the spot) very strongly depends on the source’s angular distribution, that is, on the shape and size of the plasma plume. A strongly confined plume in the case of PLD in open air causes a strongly nonuniform thickness distribution. This makes it impossible to correctly determine the sample thickness. Furthermore, the realization of the PLD setup in an oblique angle deposition geometry additionally complicates the correct measurement of sample thickness. The shadowing effect and competition between the nanocolumns during the growth leads to the fabrication of nanocolumns of different height. In this regard, a range of the sample thicknesses is presented instead of a consistent value. The thickness of the material deposited varied in the range of 540–610 nm for geometry 1, 460–750 nm for geometry 2, 410–700 nm for geometry 3, and 700–800 nm for geometry 4. The highest transparency is observed for the sample produced under geometry 1. Since the structure deposited at this geometry possesses the highest nanocolumn density, it is confirmed that the thickness of the material deposited is the lowest among all structures. The samples prepared using geometry 1, 2 and 3 also show a slight dip in the transmission spectra with minimum at ≈520 nm. The presence of this feature in the transmission could be attributed to a plasmon excitation in the nanostructures. The deposition using geometry 4 resulted in an almost flat spectrum with the lowest transmission (<3%) and no clear plasmon behavior observed. The lowest transmission of the sample is due to the highest thickness of the structure formed. Therefore, under the conditions described, the most efficient deposition of material is realized by using the on-axis configuration. It should be mentioned that the optical properties of the nanostructures considered result from the interplay of complex phenomena arising from the complex nanoparticle-ensemble morphology of the structures as no individual nanoparticles are present. The pronounced expression and definition of a plasmon resonance band is thus hindered. In such a case, the optical properties are defined by the collective effects (as multiple scattering and plasmon coupling) of an electromagnetic field interacting with nanoparticle ensembles. This leads to a broadening of the resonance band, where the optical properties of a single nanoparticle are not expressed.

To examine the conductive properties of the structures produced, we measured their electrical resistance. Table 2 shows the electrical resistance of the Au samples discussed in this study. As can be seen, the open-air laser deposition resulted in the formation of conductive structures. The resistance of the structures was measured in the range from a few ohms to several kΩ. It was found that the electrical properties of nanostructures produced by PLD in open air strongly depend on the density and morphology of the structure. The columnar structure presented in Figure 4c demonstrates an electrical resistance twice as a high as that of the sample presented in Figure 4a. In the case of geometry 2, the sample shows an even higher resistance, which is related to the mostly discrete character of the structure. The highest conductivity was measured for the sample obtained using on-axis deposition due to the dense substrate coverage. It should be mentioned that a feedback between the optical and electrical properties of the nanostructures was observed. Higher optical transmission of the nanostructures was associated with a predominantly discrete morphology, as was discussed above. The conductivity of the samples was found to increase with the accumulation of the ablated material on the substrate, which resulted in denser structures. Consequently, the implementation of in situ monitoring of the sample resistance during the PLD process would allow for an easy fabrication of nanostructures with desirable electrical and/or optical properties.

**Table 2:** Electrical resistance and hydrophobicity of the Au nanostructures produced under different geometries.

Samples	Geometry 1	Geometry 2	Geometry 3	Geometry 4

Electrical resistance [Ω]	380 Ω	1700 Ω	750 Ω	5 Ω
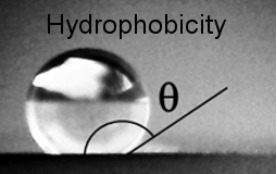	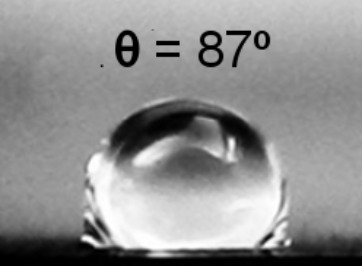	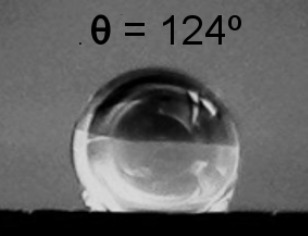	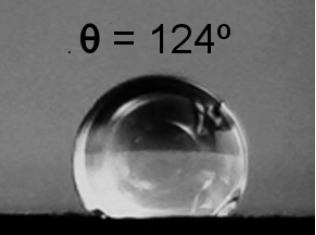	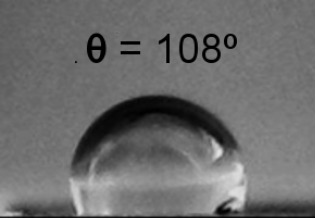

**Figure 6:**
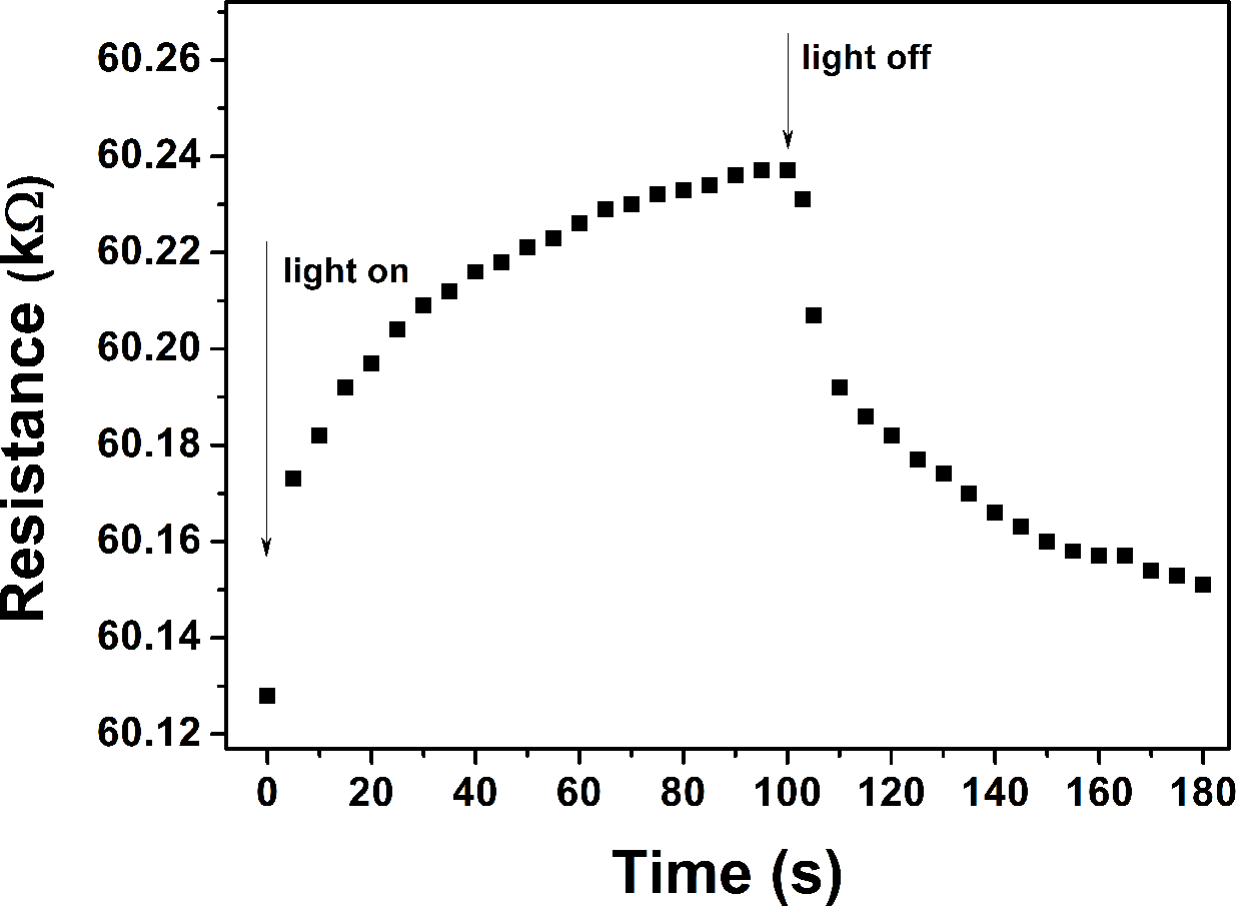
Modification of the Au nanostructure resistance by irradiation with infrared light.

The possibility of modulating the electrical properties of the nanostructures was demonstrated by irradiating them by infrared (IR) light. The change of the sample resistance upon IR irradiation is presented in Figure 6. An increase of the sample resistance was observed when the IR radiation was switched on. The change in the resistance reached several tens of ohms. A fast decrease of the resistance was initially observed when the light was switched off. Then, the rate of decrease of the resistance slowed down and reached the initial resistance value. In order to clarify the results, a thin Au film (deposited in vacuum) was also irradiated by the same IR source. No change of the resistance value of the Au film was observed during IR irradiation. The porous nanostructures grown using open-air PLD fully absorb the light radiation as a black body, which probably led to the increase in the temperature and the subsequent increase in the resistance of the structures. The smooth surface and the high reflection coefficient of the Au film do not imply light absorption. It could be concluded that the modification of the electrical properties by light irradiation is a thermal effect. Unfortunately, an adequate simulation based on thermal effects cannot be performed due to the complicated morphology and unknown thermophysical parameters values of the produced structures. It should be mentioned that the observed effect is more pronounced for the samples with electrical resistance in the range of kΩ, independent of the deposition geometry used. To the best of our knowledge, the modification of the electrical properties by light illumination has not been previously reported.

As it is well known from the literature, the porous structures exhibit good hydrophobic properties [30]. The morphology of the nanostructures produced in this study focused our attention on their hydrophobic behavior. Table 2 presents the contact angle for the samples obtained at different deposition geometries, together with the images of the droplets formed on the sample surface. All samples present a hydrophobic behavior, except for the one deposited using geometry 1, where the contact angle θ is less than 90°. The most pronounced hydrophobicity was observed for the structures shown in Figure 4b,c, where the contact angle was greater than 120°. It is known that the structures consisting of such nanocolumnar arrays possess good dynamic hydrophobicity due to their discrete surface structure [30].

## Conclusion

The present paper demonstrates a fast and flexible method for the fabrication of pure Au nanostructures. The structures were produced by applying the PLD technique in open air, that is, in the absence of vacuum, typically required for such fabrication techniques. It was found that the morphology of the as-deposited samples strongly depends on the PLD geometry. Au column-like nanostructures were produced by using different deposition geometries. The size and density of the nanocolumns was found to depend on the position of the substrate with relation to the plasma plume. The physical properties of the structures obtained by PLD in open air depend on their surface morphology. The most hydrophobic structures were those composed of distinctly separated nanocolumns, where contact angles over 120° were measured. The predominantly discrete character of these structures led to a lower electrical conductivity compared to the other samples. The accumulation of ablated material on the substrate resulted in the formation of denser structures with higher conductivity and lower transparency. The use of in situ monitoring of the sample resistance during the PLD process would allow for easy fabrication of nanostructures with engineered electrical and/or optical properties. The modification of the electrical resistance of the structures was achieved by IR light irradiation. It was found that the effect is thermal and pronounced for nanostructures only. The technique proposed is a good alternative to those already developed, as it allows for easy and simple fabrication of pure Au nanocolumn structures, avoiding the need of a low-pressure environment.
